# Influenza A/H1N1 Severe Pneumonia: Novel Morphocytological Findings in Bronchoalveolar Lavage

**DOI:** 10.1155/2014/470825

**Published:** 2014-10-14

**Authors:** Paola Faverio, Stefano Aliberti, Clinton Ezekiel, Grazia Messinesi, Ambrogio Brenna, Alberto Pesci

**Affiliations:** ^1^Department of Health Science, University of Milan Bicocca, Clinica Pneumologica, AO San Gerardo, Via Pergolesi 33, 20900 Monza, Italy; ^2^University of Texas Health Science Center at San Antonio, 8300 Floyd Curl Drive, San Antonio, TX 78229, USA; ^3^Anatomia Patologica, AO San Gerardo, Via Pergolesi 33, 20900 Monza, Italy

## Abstract

We present the results of bronchoalveolar lavage (BAL) performed in three patients with severe influenza A/H1N1 pneumonia complicated by acute respiratory distress syndrome (ARDS). Light microscopy analysis of BAL cytocentrifugates showed the presence of characteristic large, mononuclear, plasmoblastic/plasmocytoid-like cells never described before. Via transmission electron microscopy, these cells were classified as atypical type II pneumocytes and some of them showed cytoplasmic vesicles and inclusions. We concluded that plasmoblastic/plasmocytoid-like type II pneumocytes might represent a morphologic marker of A/H1N1 influenza virus infection as well as reparative cellular activation after diffuse alveolar damage.

## 1. Introduction

Several studies reported histophatological observations during influenza A/H1N1 2009 pneumonia: the major finding was diffuse alveolar damage (DAD) [1–4]. Type II pneumocytes were considered the main target of influenza A/H1N1 infection [5]. A limited number of observations described in vivo samples, such as bronchoalveolar lavage (BAL) [6–8]. BAL may be useful for cytological analysis in influenza virus pneumonias. Therefore, it may play a role in further understanding the pathogenesis of new viral strains.

In this case series we present the results of BAL performed in three patients with severe influenza A/H1N1 2009 pneumonia complicated by acute respiratory distress syndrome (ARDS).

## 2. Materials and Methods

Between August and December 2009 we performed a bronchoscopy-guided BAL of three patients admitted to the San Gerardo Hospital, Monza, Italy, for influenza A/H1N1 virus pneumonia with subsequent development of ARDS. All three patients required intensive care unit (ICU) admission and received mechanical ventilation (MV) and extracorporeal respiratory support (ECMO). Influenza A/H1N1 diagnosis was confirmed by A/H1N1 virus RNA detection on nasal swab samples using Influenza A/H1N1 2009 rRT-PCR (real-time Reverse-Transcriptase-Polymerase-Chain-Reaction) assay (Genexpert).

BAL differential cell count on cytocentrifugate was performed in the light microscopy (LM) by counting about 300 cells in random fields at 400x magnification. Morphological characteristics of cells were registered after May Grunwald Giemsa and Papanicolau staining. Additional immunocytochemical analyses were performed on two samples using CD20 and CD138. Semithin sections were prepared for transmission electron microscopy (EM) observations.

## 3. Results

Demographics, comorbidities, severity on admission, microbiological isolations, and outcome for each patient are described in Table 1.

Antiviral therapy with Oseltamivir (150 mg twice daily) was started at hospital admission simultaneously with rRT-PCR assay and was continued until rRT-PCR assay on nasal swab turned negative.

**Figure 1:**
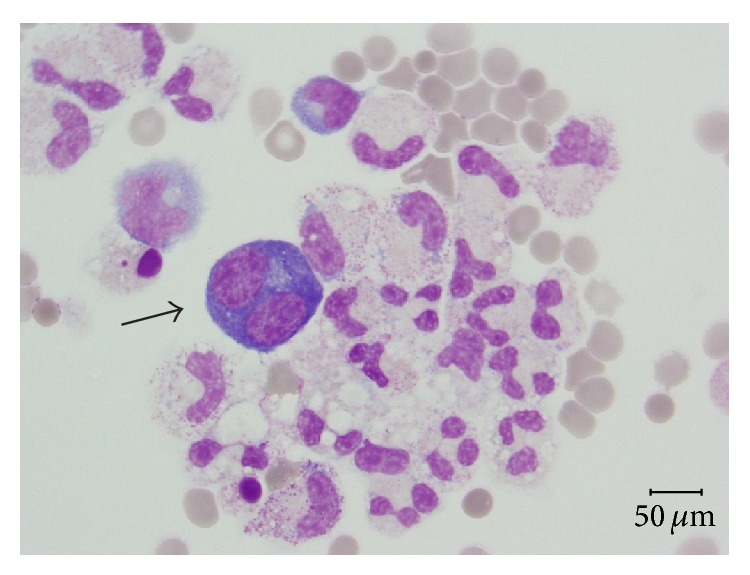
Specimen from bronchoalveolar lavage displays some macrophages, neutrophilic granulocytes, and large plasmoblastic/plasmocytoid-like elements (arrow) with basophilic cytoplasm and paranuclear vacuole (May Grunwald Giemsa, magnification ×400).

BAL cytocentrifugates analyses are summarized in Table 2. Large, atypical cells with plasmocytoid appearance were observed in all three specimens with similar percentages (8, 9, and 6%, resp.). At LM, they appeared as large, plasmoblastic/plasmocytoid-like cells with eccentric nuclei and paranuclear vacuoles, high ratio nucleus/cytoplasm, and intensely basophilic cytoplasm. No clear viral cytopathic effect was observed (Figure 1). Immunocytochemical analysis on cytocentrifugate showed that these cells were negative for CD20 (B lymphocyte marker) and CD138 (plasma cell marker).

**Figure 2:**
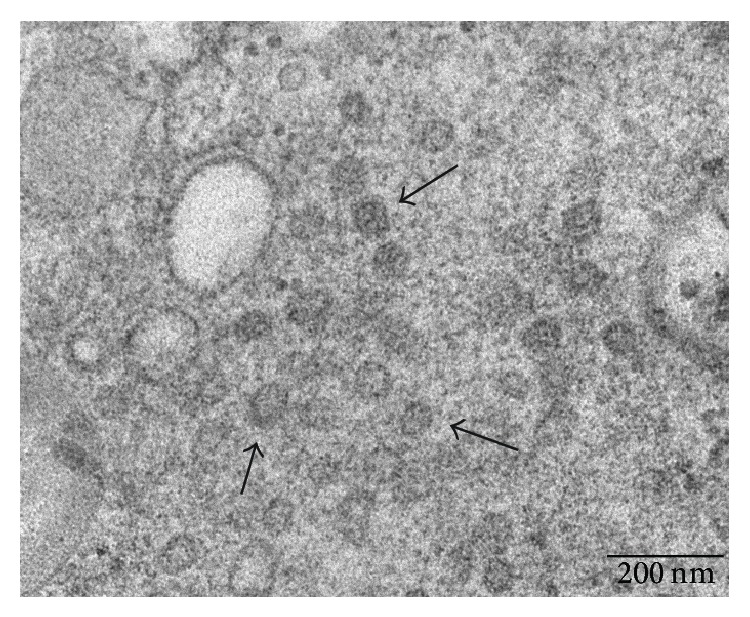
Ultrastructural examination of atypical type II pneumocytes from bronchoalveolar lavage specimen. Arrows indicate suspected cytoplasmic viral inclusions. Scale bar: 200 nm.

**Figure 3:**
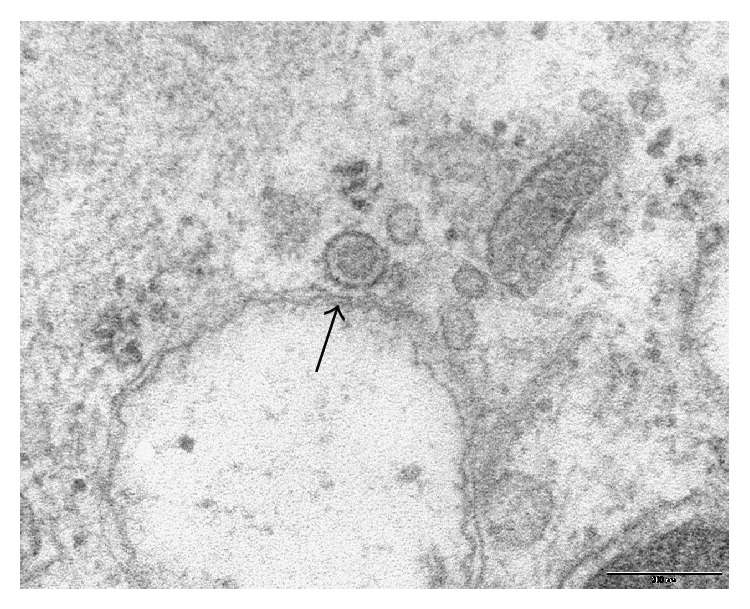
Ultrastructural examination of atypical type II pneumocytes from bronchoalveolar lavage specimen. Arrow indicates a vesicle containing a suspected viral particle. Scale bar: 200 nm.

Ultrastructural examinations at transmission EM allowed us to observe multilamellar osmiophilic bodies in the cytoplasm of plasmoblastic/plasmocytoid-like cells. Osmiophilic bodies produce surfactant and are specific for type II pneumocytes. Therefore, these cells were classified as atypical type II pneumocytes. EM showed in these cells small round cytoplasmic inclusions with irregular surface and a diameter around 60 nm (Figure 2). They also showed cytoplasmic vesicles with a diameter around 100 nm and an irregular electron-dense core (Figure 3). These aspects were suspected to be viral inclusions.

## 4. Discussion

The main characteristic of the BAL of our patients with influenza A/H1N1 pneumonia associated with ARDS was the presence of large cells with a plasmoblastic/plasmocytoid-like appearance, identified at EM as atypical type II pneumocytes.

We postulate two possible explanations for the presence of these atypical cells.

The first hypothesis regards the reparative action that type II pneumocytes may have in DAD. In our cases, type II pneumocytes activation would be justified by viral damage to alveolar epithelium. Reactive hyperplasia of type II pneumocytes is typical of the organizing stage of ARDS. Stanley described morphological atypias, such as increased nuclear-cytoplasmic ratio, in type II pneumocytes found in BALs during ARDS [10]. These atypical pneumocytes are usually aggregated in clusters and are supposed to have a reparative role.

All our patients developed ARDS and we observed pneumocytes aggregated in clusters in the BALs performed earlier (cases numbers 2 and 3). According to this first hypothesis, the atypical cells we observed could be described as reactive immature type II pneumocytes.

The second hypothesis regards influenza A/H1N1 virus cytopathic effect. Type II pneumocytes have been described as the main target of influenza A/H1N1 2009 infection [5]. Nakajima et al. in 2012 described four autopsy cases of influenza A/H1N1 with a histopathological pattern of acute DAD who presented with influenza virus antigen-positive type II pneumocytes, perhaps indicating a direct role of the virus-infected cells in the acute alveolar damage [11]. No specific cytophatic effect or viral inclusion has been described so far at LM in lung tissue specimens during influenza pneumonia [1].

Several observations have been performed via EM on autopsy specimens. During the recent influenza pandemic, Mauad et al. found type II pneumocytes with vesicles, approximately 100 nm in diameter, with an electron-dense center [2]. Bal and colleagues described cytoplasmic inclusions in pneumocytes, which ranged in diameter from 74 to 82 nm and showed surface spikes characteristic of influenza virus [12].

EM observations of our specimens revealed some atypical pneumocytes showing both small round cytoplasmic inclusions with an irregular surface and small vesicles (similar to those described by Bal and Mauad). According to this second hypothesis, atypical type II pneumocytes could be a specific morphological marker of influenza virus infection.

From the data collected so far, we cannot favor one explanation; in fact, they could coexist in cases of severe influenza A/H1N1 pneumonia associated with ARDS.

Limitations of the present study include the following: first, the paucity of cases analyzed; second, we did not perform immunohistochemical staining for H1N1 antigens on BAL samples.

The description of BAL in a control group of patients with ARDS not associated with H1N1 pneumonia is beyond the scope of this case series. However, other authors described BAL cytology in non-H1N1 ARDS, and although they reported morphological atypias in type II pneumocytes, they did not describe these peculiar plasmoblastic/plasmocytoid-like cells [10, 12, 13].

The collection of the airway specimens of patients number 2 and number 3, in which the nosocomial pathogens were isolated, took place after BAL was performed. Therefore, these pathogens most likely did not affect the cellularity in BAL.

In conclusion, plasmoblastic/plasmocytoid-like type II pneumocytes characterize the BAL of our patients with influenza A/H1N1 2009 pneumonia associated with ARDS. They could represent a pathognomonic marker of influenza virus pneumonia as well as reparative cellular activation after DAD. More observations of BAL cytology in patients with influenza pneumonia are needed to understand their characteristics and role.

## Figures and Tables

**Table 1:** Demographics, comorbidities, severity on admission, clinical course, microbiological isolations, and outcome.

	Patient 1	Patient 2	Patient 3
Age	51	43	24
Gender	Male	Female	Male
BMI	27	32	24
Immunosuppression	No	No	No
Comorbidities	Gout	Hypothyroidism, depression, chronic headache	None
SOFA score on admission	6	4	6
CURB-65 score on admission	0	1	2
Hospital day when MV was started	3rd	2nd	2nd
Hospital day when ECMO was started	7th	2nd	4th
Length of ECMO (days)	20	26	15
Hospital day when influenza virus assay was performed and oseltamivir started	3rd	2nd	1st
Length of oseltamivir therapy (days)	13	9	30
Nosocomial pathogens isolated	*C. parapsilosis* on throat specimen and *S. epidermidis* on BAS	*P. aeruginosa* MDR, *C. albicans*, *C. glabrata,* and *C. parapsilosis* on BAS	*P. aeruginosa* MDR, *Flavobacterium,* and *C. indologenes* on BAS
LP and SP urinary antigens	Negative	Negative	Negative
CP, MP, and LP IgM and IgG	Negative	Negative	Negative
Outcome	Alive	Alive	Alive

MV: mechanical ventilation; ECMO: extracorporeal membrane oxygenation; LP: *L. pneumophila*; SP: *S. pneumoniae*; CP: *C. pneumoniae*; MP: *M. pneumoniae*; BAS: bronchial aspirate; MDR: multidrug resistant (used for pathogens resistant to at least three drugs from different antibiotic categories).

**Table 2:** Cytological examination of the bronchoalveolar lavage.

	Patient #1	Patient #2	Patient #3	Reference intervals^#^
Day after hospitalization BAL was performed	15th	6th	5th	
Alveolar macrophages %	36	31	33	80–90%
Lymphocytes %	46	15	21	5–15%
Neutrophils, %	3	44	38	1–3%
Mast cells %	6	0	2	<1%
Eosinophils %	2	1	1	≤1%
p/p-like cells %	8	9	6	Absent
Red blood cells	Present	Present	Present	Absent
Type II pneumocytes aggregated in clusters	Absent	Present	Present	Absent

p/p-like cells: plasmoblastic/plasmocytoid-like cells.

^#^BAL Cooperative Group Steering Committee. Bronchoalveolar lavage constituents in healthy individuals, idiopathic pulmonary fibrosis, and selected comparison groups [9].

## References

[1] Mukhopadhyay S., Philip A. T., Stoppacher R. (2010). Pathologic findings in novel Influenza A (H1N1) virus (“Swine Flu”) infection: contrasting clinical manifestations and lung pathology in two fatal cases. *American Journal of Clinical Pathology*.

[2] Mauad T., Hajjar L. A., Callegari G. D., Da Silva L. F. F., Schout D., Galas F. R. B. G., Alves V. A. F., Malheiros D. M. A. C., Auler J. O. C., Ferreira A. F., Borsato M. R. L., Bezerra S. M., Gutierrez P. S., Caldini E. T. E. G., Pasqualucci C. A., Dolhnikoff M., Saldiva P. H. N. (2010). Lung pathology in fatal novel human influenza A (H1N1) infection. *American Journal of Respiratory and Critical Care Medicine*.

[3] Nakajima N., Hata S., Sato Y., Tobiume M., Katano H., Kaneko K., Nagata N., Kataoka M., Ainai A., Hasegawa H., Tashiro M., Kuroda M., Odai T., Urasawa N., Ogino T., Hanaoka H., Watanabe M., Sata T. (2010). The first autopsy case of pandemic influenza (A/H1N1pdm) virus infection in Japan: detection of a high copy number of the virus in type II alveolar epithelial cells by pathological and virological examination. *Japanese Journal of Infectious Diseases*.

[4] Gill J. R., Sheng Z.-M., Ely S. F., Guinee D. G., Beasley M. B., Suh J., Deshpande C., Mollura D. J., Morens D. M., Bray M., Travis W. D., Taubenberger J. K. (2010). Pulmonary pathologic findings of fatal 2009 Pandemic influenza A/H1N1 viral infections. *Archives of Pathology and Laboratory Medicine*.

[5] Shieh W.-J., Blau D. M., Denison A. M., DeLeon-Carnes M., Adem P., Bhatnagar J., Sumner J., Liu L., Patel M., Batten B., Greer P., Jones T., Smith C., Bartlett J., Montague J., White E., Rollin D., Gao R., Seales C., Jost H., Metcalfe M., Goldsmith C. S., Humphrey C., Schmitz A., Drew C., Paddock C., Uyeki T. M., Zaki S. R. (2010). 2009 Pandemic influenza A (H1N1): pathology and pathogenesis of 100 fatal cases in the United States. *The American Journal of Pathology*.

[6] Yokoyama T., Tsushima K., Ushiki A., Kobayashi N., Urushihata K., Koizumi T., Kubo K. (2010). Acute lung injury with alveolar hemorrhage due to a novel swine-origin influenza a (H1N1) virus. *Internal Medicine*.

[7] Jeon E. J., Kim K. H., Min K. H. (2010). Acute eosinophilic pneumonia associated with 2009 influenza A (H1N1). *Thorax*.

[8] Igusa R., Sakakibara T., Shibahara T., Sakamoto K., Nishimura H., Ota K. (2012). Complicated secondary pneumonia after swine-origin infuenza a virus infection in an immunocompetent patient. *Tohoku Journal of Experimental Medicine*.

[9] (1990). Bronchoalveolar lavage constituents in healthy individuals, idiopathic pulmonary fibrosis, and selected comparison groups. The BAL Cooperative Group Steering Committee. *The American Review of Respiratory Disease*.

[10] Stanley M. W., Henry-Stanley M. J., Gajl-Peczalska K. J., Bitterman P. B. (1992). Hyperplasia of type II pneumocytes in acute lung injury: cytologic findings of sequential bronchoalveolar lavage. *American Journal of Clinical Pathology*.

[11] Nakajima N., Sato Y., Katano H., Hasegawa H., Kumasaka T., Hata S., Tanaka S., Amano T., Kasai T., Chong J.-M., Iiduka T., Nakazato I., Hino Y., Hamamatsu A., Horiguchi H., Tanaka T., Hasagawa A., Kanaya Y., Oku R., Oya T., Sata T. (2012). Histopathological and immunohistochemical findings of 20 autopsy cases with 2009 H1N1 virus infection. *Modern Pathology*.

[12] Bal A., Suri V., Mishra B., Bhalla A., Agarwal R., Abrol A., Ratho R. K., Joshi K. (2012). Pathology and virology findings in cases of fatal influenza A H1N1 virus infection in 2009-2010. *Histopathology*.

[13] Ravaglia C., Gurioli C., Casoni G. (2012). Diagnostic role of rapid on-site cytologic examination (ROSE) of broncho-alveolar lavage in ALI/ARDS. *Pathologica*.

